# Postpartum Ovarian Vein Thrombosis: Incidental Diagnosis at Surgery

**DOI:** 10.1155/2014/898342

**Published:** 2014-01-12

**Authors:** Adebiyi Gbadebo Adesiyun, Modupeola O. A. Samaila, Austin Ojabo

**Affiliations:** ^1^Department of Obstetrics & Gynaecology, Ahmadu Bello University Teaching Hospital, Zaria, Nigeria; ^2^Department of Pathology, Ahmadu Bello University Teaching Hospital, Zaria, Nigeria; ^3^Department of Obstetrics & Gynaecology, Benue State University, Makurdi, Benue, Nigeria

## Abstract

Ovarian vein thrombosis is a rare clinical entity that may present in the puerperium. We report the clinical outcomes of two cases of postpartum ovarian vein thrombosis, incidentally diagnosed at laparotomy in 16-year-old and 23-year-old females. They had preoperative diagnosis of torsion tuboovarian mass and twisted pedunculated uterine fibroid, respectively. Both patients had transection and ligation of right thrombosed ovarian vein. Postoperative management included a week course of anticoagulant, antibiotics and analgesia. Postpartum ovarian vein thrombosis is a diagnosis of exclusion in the puerperium and a high index of suspicion will reduce associated morbidity and mortality.

## 1. Introduction

Postpartum ovarian vein thrombosis (POVT) is a rare puerperal complication, with an incidence of 1/600 and 1/2000 deliveries [1]. Its occurrence in nonpregnant patients has seldom been reported [2]. Three factors important in the pathogenesis of thrombosis are blood flow stasis, endothelial injury and hypercoagulability states [3]. The postpartum period is known to facilitate the occurrence of blood stasis due to collapse of the ovarian vein that was hitherto three times larger with sixty times increase in blood flow during pregnancy [3]. A physiologic hypercoagulability resulting from increased production of clotting factors I, II, VII, X and XI and increase platelet adhesiveness are also seen in the puerperium [3]. Endothelial injury is usually triggered by exogenous factors like intrauterine and urinary tract infection [3].

The aim of this paper is to present the clinical outcomes of two cases of postpartum ovarian vein thrombosis diagnosed incidentally at laparatomy.

## 2. Case 1

**Figure 1:**
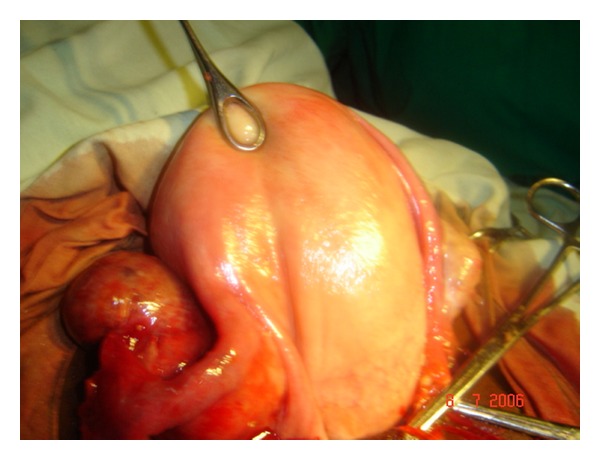
Showing bulky uterus and oedematous right adenexium that is proximal to the thrombosed right ovarian vein.

A 16-year-old para 2 female presented with 2 days history of right sided lower abdominal pain with associated fever. Five days prior to presentation, she had boy by a traditional birth attendant at home. Examination revealed an ill-looking woman with a temperature of 38.4°C. The uterus was consistent with sixteen weeks pregnancy size. Ultrasound scan showed a right tuboovarian mass that measured 7.2 cm × 6.0 cm. The uterine cavity was empty. A clinical diagnosis of right tuboovarian mass with torsion was made. Packed cell volume was 31% and there was leucocytosis of 13.0 × 10^9^/L. She had laparotomy. Intraoperatively, a thrombosed right ovarian vein was found with oedematous right adnexium (Figure 1). She had transection and ligation of the right ovarian vein. Postoperatively, she was placed on antibiotics (metronidazole and ceftriaxone), analgesia (pentazocine), and subcutaneous heparin; 5,000 units daily for a week.

## 3. Case 2

A 23-year-old para 1 female, presented with a week history of right sided lower abdominal pain and low grade fever. She had an uneventful hospital delivery four weeks prior to presentation. Her past medical history was not contributory. Clinical examination showed an ill-looking woman with a temperature of 38°C. The uterus was not palpable per abdomen; however, there was right iliac fossa tenderness with a palpable vague mass. A diagnosis of twisted ovarian mass with a differential of twisted pedunculated fibroid and complicated appendicitis was made. Her packed cell volume was 30%, platelet count was normal, and the leucocyte count was 14.1 × 10^9^/L. She was prepared for laparotomy. Intraoperatively, a cord like structure that felt woody was traced through the right infundibulopelvic fold towards the inferior vena cavae. Other pelvic structures were grossly normal. She had transection and ligation of the thrombosed right ovarian vein. Her postoperative period was uneventful. She was managed with on antibiotics (augmentin), analgesia (pentazocine), and a week course of enoxaparin injection 40 mg daily.

## 4. Discussion

Postpartum ovarian vein thrombosis (POVT) occurs in 0.05% of all pregnancies that results in live births [3] and usually follows vaginal delivery [4]. Ovarian vein is the commonest vein involved in puerperal pelvic thrombophlebitis [5]. Incidental diagnosis of POVT at surgery is not uncommon even in resource rich medical unit [4] as seen in our cases. Identified risk factors to POVT include puerperium, multiparity, postoperative period, and underlying diseases like Crohn's disease, malignant tumor, systemic lupus protein C and S, thrombophilia, and hyperhomocysteinaemia [3]. It may also complicate group A and B streptococcal infection of the vagina and endometrium [2, 6] resulting in endothelial injury.

The clinical presentation is usually within the first 4 weeks postpartum [3], and 90 percent of cases present within the first 10 days after delivery [2]. Symptoms are usually vague; however, clinical feature is characterized by fever, lower abdominal pain, and leucocytosis [2, 3]. Right ovarian vein was implicated in our patients. Studies [7] have shown that 80% of cases affect the right ovarian vein, while the left ovarian vein and both ovarian veins are involved in 6% and 14% of cases, respectively. The increase involvement of the right ovarian vein maybe explained by the compression of the inferior vena cavae and right ovarian vein due to dextrorotation of the uterus during pregnancy. Other contributory factors are antegrade flow of blood in the right ovarian vein favoring bacterial infection, in contrast to retrograde blood flow in the left ovarian vein. Also there are multiple incompetent valves in the right ovarian vein [3].

Diagnosis of POVT can be made by Doppler sonography, contrast enhanced computerized tomography scan, and magnetic resonant angiography [2]. The latter has 100 percent sensitivity and specificity. Laparoscopy is also a useful diagnostic method [8]. Appendicitis, endometritis, pyelonephritis, adnexal torsion/abscess, which are common causes of lower abdominal pain in the puerperium, should be considered as differential diagnosis [2]. In females of Black African descent, torsion of a pedunculated uterine fibroid should be included in the list of differential diagnosis of POVT.

Management approach of POVT may be medical or surgical treatment, with both recording similar success rate [3]. The main approach to medical treatment involve the use of anticoagulant [2]. The inclusion of broad spectrum antibiotics for 7 to 10 days has also been recommended [2]. While the place of surgery in the initial management of POVT is controversial [2], some clinicians prefer surgery for complicated cases associated with free floating thrombosis, recurrent pulmonary emboli in spite of medical treatment, and contraindication to anticoagulant use [2, 3]. The 2 patients managed had surgical ligation of the right ovarian vein. In cases where there is need for combined ligation of both the ovarian vein and inferior vena cavae due to thrombus extension to the latter, complications like postoperative oedema, recurrent thrombophlebitis, leg ulcers, stasis dermatitis, venous claudication, and remote mortality rate of about 15 percent may ensue [3]. Where available, the use of inferior vena cavae Greenfield filter as an alternative to ligation procedure may help decrease the incidence of complications associated with combined ovarian vein and inferior vena cavae ligation [2, 3].

Mortality rate of 52% was recorded among untreated cases [3]. However, with the use of anticoagulant, the mortality among treated cases reduced from 25% to 5%. The two patients managed had surgical ligation of the right ovarian vein and anticoagulant therapy. Recurrence of POVT is low in subsequent pregnancy [9]. But for patients with underlying hypercoagulable state, anticoagulant prophylaxis is recommended in future pregnancies [2].
